# Two Cases of Operation on the Kidney

**Published:** 1897-12-25

**Authors:** J. N. C. Davies-Colley

**Affiliations:** Surgeon to the Hospital


### Medical Progress and Hospital Clinics.

[*The Editor will be glad to receive offers of co-operation and contributions from members of the profession. All letters should be addressed to* The Editor, at the Office, 28 & 29, Southampton Street, Strand, London, W.C.]

### TWO CASES OF OPERATION ON THE KIDNEY.

Abstract of a Clinical Lecture given at Guy's Hospital by J. N. C. Davies-Colley, M.A., M.B., C.M., Surgeon to the Hospital.

(*Reported for* The Hospital, *and corrected by the Author.*)

The first case was that of a single woman, aged 39, who was admitted with a swollen abdomen, and a history of dragging pain in the right side for three years. There was no history of abnormal frequency of micturition. The swelling in the abdomen was first noticed five months ago. On admission there was an abdominal swelling situated in the right lumbar, hypogastric, and iliac regions, and not extending across the median line. There was an area of resonance between it and Poupart's ligament, and between it and the liver. The urine was clear, acid, sp. gr. 1,016, no albumen, sugar, pus, or blood, only a little mucus.

Regarding the diagnosis, it was pointed out that this was not inflammatory, because the temperature was normal; nor was it liver, because there was an area of resonance between the tumour and the liver, and the tumour was not moved by respiration. Nor was it ovary, because it did not start from the pelvis but from the lumbar region; moreover, in ovarian tumours there is resonance in the flank, which there was not here; a vaginal examination also gave negative results.

The lecturer next pointed out that, supposing this to be kidney, the question arose, What form of kidney tumour was it? It was not a sarcoma, because it was uniformly fluctuating; and there had been no blood in the urine as you would expect in sarcoma. Hydatid of the kidney would give rise to such a tumour, and the lecturer mentioned two such cases which had been recently under his care. But this affection was rare. Hydronephrosis was the most probable diagnosis, although there was no history of the occasional passage of large quantities of urine accompanied by diminution of the size of the tumour.

It was next shown how in obstruction of the ureter a ballooning out of the pelvis of the kidney and a flattening of the pyramids of Malpighi takes place, and the kidney substance consequently becomes atrophied. The causes of hydronephrosis are: (1.) Congenital causes, such as stenosis of ureter. (2.) Acquired, such as calculus impacted in the ureter, traumatic inflammation and contraction, kink of ureter, especially with moveable kidney; tumour pressing on ureter, calculus or tumour of bladder pressing on its orifice, enlarged prostate, urethral stricture, tight prepuce.

The methods of treatment are: (1.) If there is no inconvenient symptom, to leave it alone. (2.) To tap. (3.) To open and drain. (4.) To excise.

In this case chloroform was given and a trochar inserted just above the crest of the ilium and three inches back. Then 56 ounces of a clear fluid were withdrawn which contained traces of urea, and had a specific gravity of 1,002. Now if such a tumour fills again slowly, repeated tappings are advised. But this filled in four days, so a week later an incision was made three-quarters of an inch below the last rib, the cyst was incised, and it was found that very little kidney structure remained within its walls. It was here pointed out that, instead of excising, the surgeon might now sew the kidney to the abdominal wall and drain. But against this was the fact that an obstinate sinus might result, and that adhesions would form which would make a subsequent excision of the kidney more difficult. So in this case the enormously distended kidney was dissected away, and a double ligature put on the renal artery and vein, and the tumour removed. The kidney, which was shown, consisted of a large dilated pelvis, the kidney substance proper being so atrophied from pressure that there was very little of it left; the ureter was found to be very small and contracted.

As the kidney was very moveable, the disease might have arisen from a kink in the ureter, or possibly it was the result of congenital stenosis of the ureter. Since the operation the patient has passed about thirty ounces of urine per day, and has had no temperature, and has done well.

The second case was that of a healthy-looking girl, aged 15, with a history of pain in the left lumbar region and at the neck of the bladder, with frequent micturition. The urine was acid and contained pus and blood. Regarding these symptoms, it was pointed out that the presence of pus in an acid urine generally means that the pus comes from the kidney. It was also shown that in disease of the bladder, when frequent micturition is associated with the presence of pus and blood in the urine, it generally means either villous growth of the bladder, stone in the bladder, or cystitis. Regarding the differential diagnosis between these three conditions, it was pointed out that the presence of blood early, without pain until the later stages, points to the presence of villous growth of the bladder. Otherwise the case is probably one of cystitis, probably tubercular, or of stone. So to decide which of these two conditions is present, a sound should be passed. Then, if a stone is present, it is felt by the sound; but if the sound sets up great pain, and no stone is felt, then the case is probably one of tuberculous cystitis. The disease had been diagnosed as calculus of the kidney. As, however, as far back as a year ago she had suffered from painful micturition of great frequency, and the pain in her lumbar region had only come on a few months ago, it was thought to be more probable that the patient was suffering from tuberculous disease of the bladder and kidney. The patient was put under chloroform, and a cystoscope was passed into the bladder; this showed a reddish patch, which probably meant tuberculous disease of the bladder. The left kidney was then cut down upon and needled. No stone could be felt, but a small gritty piece was found at the upper part of the kidney. An incision was made into the kidney at this point, and some tuberculous masses scraped away. The wound was then syringed out with iodoform emulsion and drained. The bladder was emptied, and syringed out with iodoform emulsion.

The operation was followed by vomiting; the quantity of urine passed gradually diminished, and fourteen days later the patient died. At the post-mortem, both kidneys, the peritoneum, liver, spleen, and lungs were found infiltrated with miliary tubercle; and there was also some tuberculous ulceration in the mucous membrane of the bladder.

